# ARMS‐qPCR‐Based Detection of BRAF and TERT Promoter Mutations: A Cost‐Effective Strategy for Molecular Diagnosis of Papillary Thyroid Carcinoma

**DOI:** 10.1155/ije/8104155

**Published:** 2026-05-15

**Authors:** Yuanyuan Jia, Yajun Zhang, Dandan Sun, Jiabao Yang, Mengshi Zhou, Min Gao, Rui Li

**Affiliations:** ^1^ Research and Development Center, Xi’an Bioreal-Coming BioMed Center Co., Ltd., Xi’an, Shaanxi, China; ^2^ Department of Pathology, The Second People’s Hospital of Dingxi, Dingxi, Gansu, China

**Keywords:** ARMS-qPCR, BRAF V600E mutation, Sanger sequencing, TERT promoter mutation, thyroid papillary carcinoma

## Abstract

This study aimed to establish a rapid and sensitive method for detecting BRAF V600E, TERT C228T, and TERT C250T mutations using amplification‐refractory mutation system‐quantitative PCR (ARMS‐qPCR) combined with TaqMan probes. We systematically evaluated the assay’s accuracy, sensitivity, repeatability, and specificity in the laboratory and assessed detection performance in clinical samples, in which a total of 40 formalin‐fixed paraffin‐embedded samples (34 papillary thyroid carcinoma [PTC] tissues and 6 normal tissues) were analyzed by ARMS‐qPCR, commercial kits, and Sanger sequencing. In the laboratory, the ARMS‐qPCR assay exhibited superior performance of accuracy, sensitivity, repeatability, and specificity. In clinical samples, ARMS‐qPCR detected BRAF V600E mutation in 82.35% (28/34) and TERT C228T in 5.9% (2/34), which the commercial kit performed equally, while Sanger sequencing identified BRAF V600E in 50% (17/34) and TERT C228T in 2.9% (1/34). Simultaneously, neither method detected TERT C250T mutations. The ARMS‐qPCR method demonstrated significantly higher sensitivity than Sanger sequencing, along with advantages of rapid turnaround (2‐3 h), cost‐effectiveness, and broad applicability. These findings highlight ARMS‐qPCR as a robust, sensitive, and efficient approach for detecting BRAF and TERT promoter mutations in PTCs, offering substantial potential for clinical molecular diagnostics and personalized management of thyroid cancer.

## 1. Introduction

Thyroid cancer has become one of the top 10 cancers and poses a significant threat to the health of the population in China, with an average annual growth rate of 14.51% [1]. Histologically, it can be classified into papillary thyroid carcinoma (PTC), follicular thyroid carcinoma (FTC), anaplastic thyroid carcinoma (ATC), and medullary thyroid carcinoma (MTC) [2–4], all of which are derived from follicular epithelial cells. PTC is the most common type of thyroid cancer, accounting for over 90% of all thyroid cancer cases and showing the most significant increase in incidence. It usually remains confined to the thyroid gland but may metastasize to regional lymph nodes. Its pathogenesis is complex and is closely related to genetic factors, environmental factors, and hormonal changes [5, 6].

During the development of thyroid cancer, two signaling pathways are involved: the mitogen‐activated protein kinase (MAPK) pathway and the phosphatidylinositol 3‐kinase and protein kinase B (PI3K‐AKT) pathway. Both pathways regulate cell proliferation, differentiation, and survival [1]. The BRAF gene is located on chromosome 7. The BRAF V600E mutation, which replaces valine (V) with glutamic acid (E) at the 600th amino acid position, activates the MAPK pathway and is associated with a high incidence rate of thyroid cancer, up to 51% [5, 7–12]. Telomerase reverse transcriptase (TERT) is located on chromosome 5 and contains 16 exons. It primarily influences cancer development by maintaining telomere length to prevent cellular senescence [13–16]. There are two common hotspot mutations, C228T and C250T, with C228T being more prevalent than C250T in most cancers [17]. The incidence of TERT promoter mutations is approximately 10% in PTC, 17% in FTC, and 40% in poorly differentiated thyroid carcinoma (PDTC)/ATC [18]. Recent studies have shown that the coexistence of BRAF V600E and TERT promoter mutations is particularly associated with high‐risk clinical and pathological features in PTC [3, 14, 18–21].

Currently, a variety of techniques are available for the clinical diagnosis of thyroid cancer, including imaging examinations, laboratory tests, and pathological assessments. These primarily involve the integration of high‐resolution ultrasound, fine‐needle aspiration biopsy (FNAB), radioactive isotope scanning, and histopathology examination, combined with molecular diagnostics [22]. However, up to one‐third of thyroid nodules remain indeterminate on cytology, posing diagnostic challenges for endocrinologists and pathologists. Meanwhile, histopathological examination remains the diagnostic gold standard, and clinicians face significant challenges in accurately assessing tumor aggressiveness and recurrence risk. Molecular changes precede histological alterations, and molecular techniques have thus been widely applied in this context. Precise molecular testing for the three hotspot mutations—BRAF V600E, TERT C228T, and TERT C250T—has been shown to markedly improve the detection of malignancy, especially in Bethesda III–IV nodules, raising diagnostic sensitivity from approximately 70% to over 90% [23, 24]. Moreover, the existence or coexistence of a high allele frequency of these variants correlates strongly with aggressive tumor behavior, providing critical information for preoperative risk stratification. In this study, formalin‐fixed, paraffin‐embedded (FFPE) tissue specimens were selected for analysis because they preserve nucleic acid integrity and are readily obtainable within routine pathological workflows, and prior investigations have demonstrated that FFPE‐derived BRAF V600E detection rates are comparable to those obtained from fresh tissue, thereby ensuring both high sensitivity and specificity of the molecular assays [23, 24]. Furthermore, this assay can subsequently be adapted for use on FNAB specimens. However, existing molecular technologies, such as Sanger sequencing, digital PCR (ddPCR), and next‐generation sequencing (NGS), vary in detection precision [19]. Sanger sequencing, while highly specific, is less sensitive and is suitable for detecting known mutations [25]. ddPCR is appropriate for detecting low‐abundance mutations but is costly [26]. NGS, due to the complexity of gene sequences, requires optimized experimental design to improve detection accuracy [27]. Traditional immunohistochemistry (IHC) is highly sensitive, but results may be affected by sample processing and antibody specificity [27]. Therefore, there is an urgent need for more advantageous methods to enhance the sensitivity of mutation detection. Amplification‐refractory mutation system‐quantitative PCR (ARMS‐qPCR) is a highly sensitive and specific molecular detection technique that can accurately distinguish between mutant and wild‐type genes, making it particularly suitable for detecting low‐abundance mutations. Compared with traditional PCR, ARMS‐qPCR is simpler, faster, and more accurate in quantitative analysis, with lower costs. It has been widely used in precision diagnostics and monitoring of cancers and genetic diseases. ARMS‐qPCR, which combines ARMS technology with real‐time quantitative PCR (qPCR), has demonstrated advantages in the sensitive detection of mutant genes.

In this study, we proposed to develop an ARMS‐qPCR assay integrating allele‐specific primers with TaqMan probes, enabling rapid (2 h), cost‐effective, and ultrasensitive detection of BRAF V600E and TERT C228T/C250T mutations. Besides, this study validates the clinical performance of ARMS‐qPCR against commercial kit and Sanger sequencing in archival FFPE thyroid specimens, with a focus on its potential to resolve diagnostic uncertainties in indeterminate nodules and stratify high‐risk PTC cases harboring concurrent BRAF/TERT mutations. By overcoming the sensitivity constraints of conventional methods, this approach could enhance preoperative risk assessment and guide personalized therapeutic strategies in thyroid cancer management.

## 2. Materials and Methods

### 2.1. Patients and Methods

**FIGURE 1:**
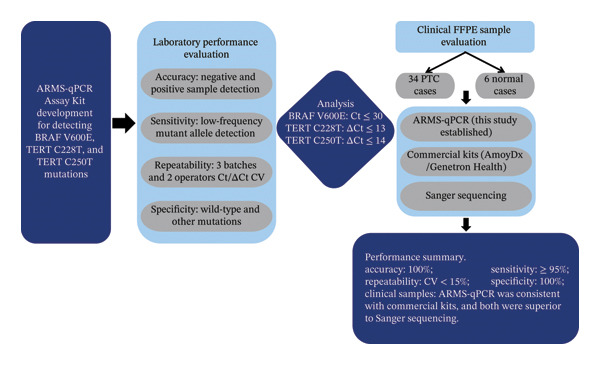
Development and evaluation workflow for the ARMS‐qPCR assay to detect BRAF and TERT promoter mutations.

A total of 40 FFPE tissue samples were collected from the Second People’s Hospital of Dingxi, comprising 34 histologically confirmed PTC cases and 6 adjacent normal thyroid tissues. All subjects gave their informed consent for inclusion before they participated in the study. The study was conducted in accordance with the Declaration of Helsinki, and the protocol was approved by the Ethics Committee of the Second People’s Hospital of Dingxi. Genomic DNA was extracted using the DNA extraction kit for paraffin‐embedded tissues (TIANGEN Biotech, DP331). PCR amplification utilized MolTaq DNA Polymerase (Novozyme, P402) on ABI 7500 and QuantStudio 5 Real‐Time PCR Systems (Thermo Fisher Scientific). Mutation detection employed allele‐specific primers and TaqMan probes (Sangon Biotech) targeting BRAF V600E, TERT C228T, and C250T. Reference standards included BRAF V600E, TERT C228T, and C250T mutant gDNA (Kebai Biotechnology). The results were validated against commercial kits (AmoyDx Diagnostics BRAF V600E Kit; Genetron Health TERT Promoter Kit) and Sanger sequencing. Specificity controls included synthetic plasmids (V600R, TERT 228M, and TERT 250M; Sangon Biotech). The overall workflow is detailed in Figure 1.

### 2.2. ARMS‐qPCR Assay Design

Primers and probes (Table 1) were designed to discriminate mutant alleles using locked nucleic acid (LNA)–modified primers for enhanced specificity. Three independent 25 μL reaction systems were optimized: BRAF V600E (1 μL MolTaq DNA Polymerase, 2.5 μL 10 × PCR Buffer, 1 μL dNTP Mix, 1 μL BRAF V600E mutated F/R primers (10 μM), 1 μL probe (2 μM), 0.5 μL internal reference F/R primers (10 μM), 1 μL (2 μM), 2 μL Mg^2+^, 1 μL DNA), TERT C228T/C250T (5 μL 5 × PCR enhancer, 1 μL MolTaq DNA Polymerase, 2.5 μL 10 × PCR Buffer, 1 μL dNTP Mix, 2μL TERT C228T/C250T mutated F/R primers (10 μM), 2 μL probe (2 μM), 0.5 μL internal reference F/R primers (10 μM), 1 μL (2 μM), 2 μL Mg^2+^, 2 μL DNA). Thermal cycling was 95°C for 15 min; 15 cycles of 95°C/15 s, 60°C/30 s; 30 cycles of 95°C/15 s, 56°C/30 s (fluorescence acquisition).

**TABLE 1:** Primer and probe sequences for BRAF V600E, TERT C228T/C250T, and internal reference.

Target	Primer/probe	Sequence (5′ ⟶ 3′)	Fragment length (bp)
BRAF V600E	Forward	GTGATTTTGGTCTAGCTAC AC[LNA‐A]	149
	Reverse	TCT​AGT​AAC​TCA​GCA​GCA​TCT​CAG	
	Probe	VIC‐ATGGAGTGGGTCCCATCAGTT TGAACAG‐BHQ2	

TERT C228T	Forward	CCG​GGC​TCC​CAG​TGG​ATT​C	184
	Reverse	GCTGGGAGGGCCCGGCA	
	Probe	FAM‐CGGGCACAGACGC CCAGGACCG‐BHQ1	

TERT C250T	Forward	CCG​GGC​TCC​CAG​TGG​ATT​C	162
	Reverse	TGGGCCGGGGACCCGTA	
	Probe	FAM‐CGGGCACAGACG CCCAGGACCG‐BHQ1	

Internal reference	Forward	CGC​GAA​CTC​ACC​CGT​TGA​CT	97
	Reverse	CAC​TAG​GCG​CTC​ACT​GTT​CTC	
	Probe	CY5‐ACCTTCACCTTCCCCA TGGTGTCTG‐BHQ2	

### 2.3. ARMS‐qPCR Assay

The analytical performance of the ARMS‐qPCR assay was rigorously evaluated using three independent reagent batches produced under a certified quality management system. The analytical accuracy, sensitivity, repeatability, and specificity of the ARMS‐qPCR assay were systematically evaluated. For accuracy, negative concordance was assessed using wild‐type gDNA (10 ng/μL), while positive concordance was determined using wild‐type gDNA (10 ng/μL) spiked with BRAF V600E, TERT C228T, or C250T mutant gDNA at 20%, 12%, and 2% allele frequencies. Sensitivity was tested by two approaches: (1) detecting mutant alleles at varying proportions (5%–0.5%) in a fixed DNA concentration (10 ng/μL) and (2) determining the minimum detectable DNA concentration (5–30 ng/μL) at 1% (BRAF V600E/C250T) or 0.5% (TERT C228T) mutant allele frequencies. Repeatability was evaluated by analyzing intra‐ and interbatch precision across three independently produced reagent batches using mixed samples (BRAF V600E: 5% and 2% at 5 ng/μL wild‐type gDNA; TERT C228T: 5% and 1% at 10 ng/μL wild‐type gDNA; TERT C250T: 5% and 2% at 5 ng/μL wild‐type gDNA), with coefficients of variation (CV) calculated from 20 consecutive days of testing. Specificity was validated against wild‐type gDNA (30 ng/μL) and cross‐reactivity assessed using plasmids encoding BRAF V600R, TERT 228M, and TERT 250M mutants in a wild‐type genomic DNA background.

### 2.4. Commercial Kit Detection

The BRAF V600E mutation was detected and analyzed using the AmoyDx Diagnostics BRAF V600E Kit, while the TERT promoter variants C228T and C250T were examined with the Genetron Health TERT Promoter Kit. All procedures and result interpretations were performed strictly in accordance with the respective product manuals.

### 2.5. Sanger Sequencing

DNA extracted from FFPE tissues was amplified by PCR using specific primers for BRAF V600E (forward: 5′‐CAG​GAG​TGC​CAA​GAG​AAT​ATC​TGG‐3′; reverse: 5′‐TGG​CTG​TGG​ATC​ACA​CCT​GC‐3′) and TERT promoter regions (forward: 5′‐CGT​CCG​GCA​TTC​GTG​GTG‐3′; reverse: 5′‐CCA​CCA​GCG​CGC​GGA​AAG‐3′). Amplified products were purified and subjected to bidirectional Sanger sequencing (Sangon Biotech, Shanghai) using the forward primers (BRAF‐F and TERT228/250‐F) for mutation analysis. Sequencing chromatograms were analyzed with BioEdit Sequence Alignment Editor and aligned against reference sequences (BRAF: NG_007873.3; TERT: NG_055467.1) to confirm BRAF V600E and TERT promoter mutations (C228T and C250T) by MEGA 7.0.26.

## 3. Results

### 3.1. Demographic and Clinical Characteristics

This study included 40 individuals of 34 PTC cases and 6 normal tissues from Gansu, China (Table 2). The mean age ± standard deviation of PTC participants was 52.6 ± 10.1 years, ranging from 25 to 76 years. The majority were females (24 out of 34), with the proportion of 70.59%. Immunohistochemical confirmation was achieved in 22 cases (64.71%), with characteristic markers (e.g., CK19 and galectin‐3) demonstrating diagnostic consistency with histopathological findings.

**TABLE 2:** Demographic and clinical characteristics of study subjects.

Patients	Sex	Age	Pathologic diagnosis	Immunohistochemical
1	Female	56	Right PTC	CK19 (+), HBME‐1 (−), galectin‐3 (+), Ki‐67 (5%), P53 (+ nonsense mutation)
2	Female	56	Left PTC, diameters of 1.0 cm	/
3	Male	50	Left PTC, diameters of 1.0 and 1.2 cm	/
4	Male	56	Right PTC, diameter of 3.5 cm	/
5	Female	50	Left PTC, diameter of 1.0 cm	/
6	Female	30	Right PTC, diameter of 2.2 cm	CK19 (+), TTF‐1 (+), Tg (+), Ki‐67 (+5%)
7	Female	68	Left PTC, diameter of 1 and 0.3 cm; right PTC, diameter of 1.2 cm	CK19 (+), galectin‐3 (+), TTF‐1 (+), Tg (+), TPO (−), CD56 (part+), BRAF (±), Ki‐67 (+5%)
8	Female	63	Right PTC, diameter of < 1 cm	Tg (+), TPO (+), TTF‐1 (+), CK19 (+), galectin‐3 (+), CD56 (+), BRAF (+), Ki‐67 (< +5%)
9	Male	59	Left PTC, diameter of 1.4 cm	Tg (+), TPO (−), TTF‐1 (+), CK19 (+), galectin‐3 (+), CD5 (−), CK7 (+), BRAF (−), Ki‐67 (< +5%)
10	Female	66	Left PTC, size of 2 × 1.7 × 1.2 cm	CK19 (+), TTP‐1 (+), CEA (−), CeA (−), CKP (+), Ki‐67 (−)
11	Male	25	Left PTC, diameter of 0.7 cm	TTF‐1 (+), CK19 (+), Ki‐67 (−), Tg (+), CK7 (+), CK20 (−), CgA (−), galectin‐3 (+)
12	Female	54	Right PTC	Tg (part+), TPO (±), TTP‐1 (+), CK19 (+), galectin‐3 (+), CD56 (−), CK7 (+), Ki‐67 (<+10%)
13	Female	40	Thyroid lobes, isthmus and central lymph node, PTC, diameter of 1 cm	CK19 (+), Tg (+), TTF‐1 (+), CgA (−), CKL (+), Ki‐67 (−), EMA (−), galectin‐3 (+), CD56 (−), CEA (−)
14	Female	48	Left PTC, diameter of 0.5 cm; right PTC, diameter of 0.8 cm	Left, CK19 (+), Tg (+), TTF‐1 (+), TPO (−), galectin‐3 (+), BRAF (+), CK7 (+), CK20 (−), Ki‐67 (+< 2%); right, CK19 (+), Tg (+), TTF‐1 (+), TPO (−), galectin‐3 (+), BRAF (+), CK7 (+), CK20 (−), Ki‐67 (+2< %)
15	Female	51	PTC (classic type)	CK19 (+), galectin‐3 (+), HBME‐1 (+), CD56 (−), P53 (−), BRAF V600E (+), TROP2 (3+, 90%), Ki‐67 (10%+)
16	Female	54	PTC (classic type)	CK19 (+), galectin‐3 (+), HBME‐1 (+), CD56 (−), p53 (−), Ki‐67 (5%+)
17	Male	49	PTC (classic type)	CK19 (+), Galectin‐3 (+), HBME‐1 (+), CD56 (−), p53 (−), Ki‐67 (5%+)
18	Male	54	PTC (classic type)	/
19	Female	53	PTC (classic type)	CK19 (+), HBME (+), CD56 (−), TTF‐1 (+)
20	Female	76	PTC (classic type)	/
21	/	/	PTC (classic type)	/
22	Female	49	PTC (classic type)	Tg (+), CK20 (−), CK19 (+), TPO (+), TTF‐1 (+), CD56 (−), CgA (+), Syn (+), Ki‐67 (10%+)
23	Female	50	PTC (classic type)	Tg (+), CK20 (−), CK19 (+), TPO (+), TTF‐1 (+), CD56 (−), CgA (+), Syn (+), Ki‐67 (5%+)
24	Female	59	PTC (classic type)	Tg (+), CK20 (−), CK19 (+), TPO (+), TTF‐1 (+), CD56 (−), CgA (+), Syn (+), Ki‐67 (5%+)
25	Female	52	PTC (classic type)	Tg (+), CK20 (−), CK19 (+), TPO (+), TTF‐1 (+), CD56 (−), CgA (−), Syn (+), Ki‐67 (10%+)
26	Male	56	PTC (classic type)	Tg (+), CK20 (−), CK19 (+), TPO (+), TTF‐1 (+), CD56 (−), CgA (−), Syn (−), Ki‐67 (5%+)
27	Female	58	PTC (classic type)	Tg (+), CK20 (−), CK19 (+), TPO (+), TTF‐1 (+), CD56 (−), CgA (−), Syn (+), Ki‐67 (5%+)
28	Female	41	PTC (classic type)	Tg (+), CK20 (−), CK19 (+), TPO (+), TTF‐1 (+), CD56 (−), CgA (−), Syn (−), Ki‐67 (5%+)
29	Female	57	Papillary thyroid microcarcinoma	/
30	Male	57	PTC (classic type)	/
31	Female	46	PTC (classic type)	/
32	Female	37	PTC (classic type)	/
33	Male	60	PTC (classic type)	/
34	Female	56	PTC (follicular variant)	Tg (+), CK20 (−), CK19 (+), TPO (+), TTF‐1 (+), BRAF (+), Ki‐67 (5%+)

### 3.2. Analytical Accuracy Validation

The analytical accuracy of the ARMS‐qPCR assay was validated using negative samples and positive samples of three mutant allele frequencies. The results demonstrated 100% negative concordance (0/20 negatives) for wild‐type gDNA (10 ng/μL) across BRAF V600E, TERT C228T, and C250T detection systems. Positive concordance was also 100% (20/20 positives) for all three targets at mutant allele frequencies of 20%, 12%, and 2%. Both the negative and positive detections showed pretty good accuracy.

### 3.3. Analytical Sensitivity Validation

The BRAF V600E assay reliably detects low‐frequency mutant alleles in diluted DNA. Wild‐type genomic DNA (10 ng/μL) was mixed with BRAF V600E mutant DNA at 5%, 2%, 1%, and 0.5% mutant allele fractions; each mixture was tested in 20 independent replicates, yielding detection rates ≥ 95% and confirming reliable detection down to 1% when 10 ng of total DNA is used. Maintaining a fixed 1% mutant fraction, the total DNA input was varied to 30, 20, 10, and 5 ng/μL; all four input levels also achieved ≥ 95% detection, demonstrating consistent identification of a 1% BRAF V600E allele with as little as 5 ng DNA. The TERT C228T assay showed comparable performance: With 10 ng/μL wild‐type DNA, mutant allele fractions of 5%, 2%, 1%, and 0.5% were each detected in ≥ 95% of 20 replicates, establishing a detection limit of 0.5% at 10 ng input. Fixing the mutant fraction at 0.5% and varying DNA input to 30, 20, 10, and 5 ng/μL again produced ≥ 95% detection at the 30, 20, and 10 ng levels, confirming stable detection at the lowest input of 10 ng (≈15 mutant copies per reaction). Using the same approach, the TERT C250T assay detected mutant fractions of 5%, 2%, and 1% in 20 replicates, and with a fixed 1% fraction reliably identified the mutation at inputs of 30, 20, 10, and 5 ng/μL, achieving ≥ 95% detection even with 5 ng DNA. In a word, the BRAF V600E assay reliably detects mutant alleles present at a frequency as low as 1% in samples containing only 5 ng of DNA, the TERT C228T assay achieves stable detection of a 0.5% mutant allele frequency in 10 ng DNA samples, and the TERT C250T assay similarly detects a 1% mutation frequency in 5 ng DNA. Collectively, these validations demonstrate that the assays are robust tools for identifying clinically actionable mutations in low‐abundance specimens.

### 3.4. Analytical Repeatability Validation

To evaluate the robustness of the multiplex detection system, inter‐ and intra‐assay precision was rigorously assessed using three independent reagent batches over a 20‐day period. The analysis included mutant allele frequencies spanning clinically relevant ranges: high‐concentration (5%) and low‐concentration (1%–2%) synthetic samples for BRAF V600E, TERT C228T, and TERT C250T mutations. All mutations demonstrated 100% concordance across replicates under all tested conditions. Notably, BRAF V600E and TERT C250T showed 100% detection rate for both 5% and 2% allele frequencies. The TERT C228T promoter mutation also showed 100% sensitivity at 5% and 1% alleles. In addition, cycle threshold (Ct) variability was quantified as the CV for high‐concentration samples (5% mutant alleles), and positivity was defined as Ct ≤ 30 for BRAF V600E, ΔCt ≤ 13 for TERT C228T, and ΔCt ≤ 14 for TERT C250T. For interbatch precision, both platforms demonstrated acceptable interbatch precision (CV ≤ 15.0% for BRAF V600E and ΔCt CV ≤ 15.0% for TERT C228T/C250T) across all assays (Table 3). On the ABI 7500 platform, the BRAF V600E assay exhibited mean values of 20.17 (Operator 1) and 20.33 (Operator 2), with CVs of 3.14% and 3.49%, respectively. For TERT promoter mutations, the C228T assay showed mean ΔCt values of 6.82 (Operator 1) and 6.80 (Operator 2), corresponding to ΔCt CVs of 8.96% and 9.19%. Similarly, the C250T assay yielded mean ΔCt values of 7.70 and 7.62 for both operators, with ΔCt CVs of 10.40% and 10.78%. In contrast, the Q5 platform displayed marginally higher variability in TERT assays. The BRAF V600E assay maintained comparable precision, with mean values of 19.82 (Operator 1) and 19.90 (Operator 2) and CVs below 2.72%. However, the TERT C228T assay showed increased ΔCt CVs (12.38%–12.65%) at mean ΔCt values of 5.68 (Operator 1) and 5.82 (Operator 2). A similar trend was observed for the TERT C250T assay, with mean ΔCt values of 6.41–6.50 and ΔCt CVs ranging from 12.46% to 13.28% (Table 3). Meanwhile, intrabatch precision across three consecutive batches is summarized in Table 4. For the ABI 7500 platform, CVs for BRAF V600E ranged from 2.95% to 3.67%, while TERT assays (C228T and C250T) showed ΔCt CVs between 4.32% and 10.92%. Batch‐to‐batch consistency was maintained, with all CVs remaining below 15%. The Q5 platform achieved robust intrabatch reproducibility for BRAF V600E (CVs: 1.88%–3.31%), whereas TERT assays exhibited moderately elevated variability. The C228T and C250T assays displayed ΔCt CVs of 6.76%–13.76% across batches, with no significant differences between operators. Thus, both platforms met predefined precision criteria (CV ≤ 15%) and demonstrated comparable robustness for mutation quantification, suggesting their suitability for routine clinical use.

**TABLE 3:** Interbatch repeatability analysis of three batches of reagents on two compatible instruments using ARMS‐qPCR.

Mutations		BRAF V600E		TERT C228T		TERT C250T	
Operator		1	2	1	2	1	2
ABI 7500	MN	20.17	20.33	6.82	6.8	7.7	7.62
	SD	0.633	0.71	0.611	0.624	0.801	0.821
	CV	3.14%	3.49%	8.96%	9.19%	10.40%	10.78%

Q5	MN	19.82	19.9	5.68	5.82	6.41	6.5
	SD	0.54	0.462	0.703	0.737	0.799	0.863
	CV	2.72%	2.32%	12.38%	12.65%	12.46%	13.28%

*Note:* MN, mean value (Ct or ΔCt).

Abbreviations: CV, coefficient of variation; SD, standard deviation.

**TABLE 4:** Intrabatch repeatability analysis of three batches of reagents on two compatible instruments using ARMS‐qPCR.

Mutations		BRAF V600E		TERT C228T		TERT C250T	
Operator		1 (%)	2 (%)	1 (%)	2 (%)	1 (%)	2 (%)
ABI 7500	Batch 1	3.38	3.67	4.32	4.94	8.79	9.61
	Batch 2	2.95	3.66	6.67	6.70	7.95	8.12
	Batch 3	3.07	3.18	4.50	5.43	10.92	8.32

Q5	Batch 1	2.23	2.63	9.07	8.07	11.92	12.24
	Batch 2	2.54	1.88	8.97	9.67	7.45	10.29
	Batch 3	3.31	2.26	8.77	6.76	13.76	10.40

### 3.5. Analytical Specificity Validation

No cross‐reactivity was observed in wild‐type gDNA (30 ng/μL), with all targets showing negative signals (0/20 negative). To further validate assay specificity, homologous mutant variants (e.g., BRAF V600R, TERT 228M, and TERT 250M) were analyzed in parallel. None of these variants produced detectable amplification signals in their respective detection channels. These findings collectively confirm the absence of cross‐reactivity or nonspecific amplification under the tested conditions.

### 3.6. Detection in Clinical Specimens

**FIGURE 2:**
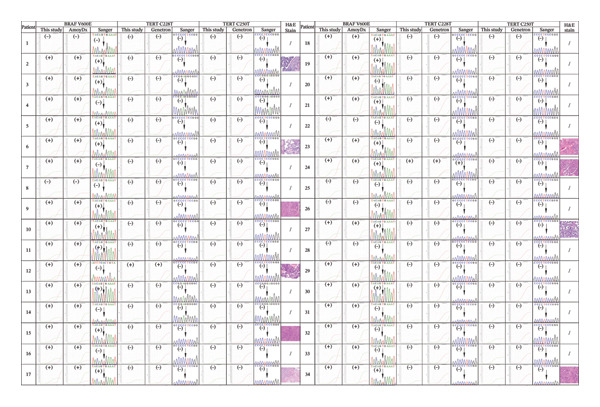
ARMS‐qPCR, commercial kit, Sanger sequencing, and H&E staining results of the 34 PTC specimens.

The ARMS‐qPCR assay we established demonstrated superior sensitivity for low‐abundance mutations across a cohort of 40 FFPE‐processed thyroid specimens (34 PTCs and 6 normal tissues). BRAF V600E mutation was identified in 82.35% (28/34) of PTCs, while TERT C228T variants were detected in 5.9% (2/34) of cases. Notably, no TERT C250T mutations were observed in any specimen, and all normal tissues showed negative results for the three targets (0/6). The assay exhibited 100% concordance with National Medical Products Administration (NMPA)‐approved diagnostic kits (AmoyDx/Genetron Health). Additionally, Sanger sequencing exhibited markedly reduced detection capability, identifying BRAF V600E in only 50% (17/34) of mutation‐positive PTCs, TERT C228T in 2.9% (1/34) of cases, and failing to detect any TERT C250T variants (0/34), despite maintaining 100% specificity in normal controls. This performance gap was particularly evident for TERT promoter mutations, where variant allele frequencies (VAFs) consistently fell below Sanger’s empirically validated 15%–20% detection threshold. The ARMS‐qPCR assay, Sanger sequencing, commercial kits, and H&E stain results of PTCs are shown in Figure 2, in which the H&E‐stained sections of microscopic evaluation revealed definitive neoplastic features, including nuclear atypia (enlarged hyperchromatic nuclei and irregular nuclear contours) and architectural disorganization, which were pathognomonic of carcinoma progression. All the results underscore ARMS‐qPCR’s enhanced diagnostic utility for molecular stratification of thyroid malignancies, particularly in FFPE‐derived DNA where fragmentation and low tumor purity frequently compromise conventional sequencing approaches.

## 4. Discussion

In recent years, the advancement of molecular biology techniques has significantly propelled the development of molecular research on thyroid cancer. In particular, BRAF V600E and TERT promoter mutations are closely associated with recurrence, mortality, and prognosis in thyroid cancer, making them hotspots in thyroid cancer research [10]. The BRAF V600E mutation is highly prevalent in PTC (with a mutation rate exceeding 80%) and is strongly correlated with poor prognosis, making it an important focus in thyroid cancer research [28–30]. TERT promoter mutations occur at a lower frequency in PTC (1.1%–4.7%), and these mutations are significantly associated with the aggressiveness and recurrence risk of thyroid cancer, particularly in advanced and highly invasive cases [30]. Moreover, the coexistence of BRAF V600E and TERT promoter mutations typically indicates a worse prognosis [31–33]. Research on these molecular markers not only provides new perspectives for the diagnosis of thyroid cancer, but also offers important references for optimizing clinical management and treatment strategies [34].

The novelty of this work lies in the development of a highly specific and low‐input ARMS‐qPCR assay for the three mutation targets BRAF V600E, TERT C228T, and C250T. Using only 5 ng of genomic DNA for BRAF V600E and TERT C250T, the assay achieves ≥ 95% detection with a limit of detection (LOD) of 1%. For TERT C228T, 10 ng of DNA yields an LOD of 0.5% (≈15‐20 mutant copies). Compared with previously reported ARMS‐qPCR methods, which typically reach an LOD of 0.5%–1% with 5–10 ng DNA [35, 36], and with most ARMS‐qPCR reports that cite a −10% detection threshold due to the GC‐rich promoter region [37], our assay provides comparable or superior sensitivity while requiring markedly less input material. In clinical validation, we tested 40 FFPE thyroid specimens (34 PTC and 6 normal) from Gansu and obtained 100% concordance with two NMPA‐approved commercial kits—AmoyDx Thyroid Panel (LOD = 1% VAF, ≥ 10 ng DNA) and Genetron Health ThyroSeq (LOD = 1% VAF, ≥ 10 ng DNA). Within the same cohort, BRAF V600E was detected in 82.35% (28/34) of cases, markedly higher than the 50% (17/34) detection rate by Sanger sequencing, and TERT C228T was reliably identified down to a VAF of 0.5%, addressing the gap in low‐frequency mutation detection in low‐quality samples. This is the first systematic evaluation of a three‐target ARMS‐qPCR on FFPE thyroid tissue from Gansu, demonstrating advantages in cost, sensitivity, and operational simplicity equivalent to commercial kits and superior to conventional Sanger sequencing, also providing a feasible molecular diagnostic solution for regional hospitals.

Compared with currently mainstream mutation detection methods, ARMS‐qPCR we established demonstrates distinct advantages in sensitivity, turnaround time, cost, and operational simplicity. With BRAF V600E and TERT C250T at a 1% VAF, and TERT C228T down to 0.5%, it markedly surpassed conventional Sanger sequencing, which is stable only above 15% VAF and yields ambiguous results between 5% and 15% [38]. Compared with NGS (LOD 0.1%–1%) but requiring extensive library preparation and sequencing time [39], and ddPCR (sub‐0.1% LOD) yet high instrument and reagent costs [26, 40], our method retains comparable or superior sensitivity while dramatically reducing material requirements. In terms of turnaround, ARMS‐qPCR completes the entire workflow within a few hours; Sanger typically needs 2‐3 days and NGS 5–10 working days, and although ddPCR can also finish in 3–5 h, its pre‐analytical steps are more cumbersome. Cost‐wise, ARMS‐qPCR offers moderate reagent expenses and low per‐sample cost, suitable for medium‐throughput routine laboratories; Sanger’s low cost applies only to small batches and escalates rapidly with multigene panels, whereas NGS and ddPCR incur high overall expenses due to expensive equipment and consumables. Considering all these aspects, ARMS‐qPCR emerges as the preferred method for detecting these specific mutations, delivering reliable results quickly and economically, thereby substantially enhancing molecular diagnostic capacity in regional and primary healthcare laboratories.

This study employed FFPE surgical resection specimens because FFPE preserves the complete tumor architecture and yields sufficient, relatively high‐quality DNA/RNA, which is essential for the high‐sensitivity detection of low‐frequency mutations; moreover, FFPE is regarded as the gold standard for tissue‐based pathology, faithfully reflecting the tumor’s in vivo biological state and providing a reliable foundation for correlating molecular alterations with invasiveness and long‐term prognosis [41, 42]. In contrast, FNAB specimens are primarily used for preoperative diagnosis, offering advantages of simplicity, minimal invasiveness, low cost, and rapid acquisition of cellular material under local anesthesia, and are widely applied to differentiate benign from malignant thyroid, breast, and other suspicious lesions [43, 44]. However, the limited cellular yield of FNA can lead to insufficient material or false‐negative results. Therefore, we first performed methodological validation on FFPE resection specimens to ensure that the assay meets the stringent performance criteria required for clinical application. Subsequently, the method can be extended to FNAB sample studies in the future.

By analyzing the BRAF V600E, TERT C228T, and TERT C250T mutations in paraffin‐embedded tissue samples from patients diagnosed with thyroid cancer and normal tissues, and comparing the results with those of Sanger sequencing, we found that ARMS‐qPCR had significantly higher detection rates for BRAF V600E and TERT C228T mutations than Sanger sequencing, with rates of 82.35% vs. 50% and 5.9% vs. 0%, respectively. In 2013, Huang et al. established an ARMS‐qPCR method that detected the V600E mutation in 67% of 33 PTC patients and in 75% of 12 PTC patients with tall cell variants. Sanger sequencing detected only 27 of the 30 mutations identified by the ARMS‐qPCR [25]. In 2022, Lu et al. analyzed the BRAF V600E mutation in 119 PTC patients using ddPCR and Sanger sequencing, with mutation detection rates of 67.23% and 26.05%, respectively. Compared with ddPCR and Sanger sequencing, the ARMS‐qPCR method established in this study demonstrated higher detection rates for BRAF V600E mutations, enhancing its broad applicability in clinical settings. Additionally, among 34 patients diagnosed with PTC, ARMS‐qPCR detected 28 BRAF V600E mutations and 2 TERT C228T mutations, whereas Sanger sequencing failed to detect 17 BRAF V600E mutations and 1 TERT C228T mutation. In addition, the detection results indicate that when ARMS‐qPCR detects the BRAF V600E mutation, a lower Ct value (around Ct 17) corresponds to a higher mutant DNA content, allowing Sanger sequencing to successfully detect the mutation. In contrast, when ARMS‐qPCR detects higher Ct values (for BRAF V600E) or ΔCt values (for TERT C228T and C250T mutations), the low mutant DNA content falls below the detection limit of sequencing, rendering Sanger sequencing unable to detect these mutations. Therefore, when the quality and quantity of DNA are at low level, ARMS‐qPCR maximizes the number of analyzable samples, improving the sensitivity and speed of detection compared with sequencing [35].

However, there are also some limitations. The clinical application involved a relatively small sample size, covering only paraffin‐embedded tissue samples from patients diagnosed with PTC and normal tissues. Future studies should expand the sample size to include more patients with different types of thyroid cancer and other related diseases, while also considering factors, such as ethnicity and geography, to comprehensively evaluate the performance of ARMS‐qPCR in various clinical scenarios [45]. In the future, we also plan to conduct a large‐scale, prospective, multicenter validation study enrolling ≥ 200 cases, to comprehensively assess the performance of the ARMS‐qPCR assay across different populations and clinical settings. Moreover, although the technology demonstrated high sensitivity, its specificity has not been thoroughly investigated. Further validation is needed to confirm its detection accuracy in complex clinical samples, to rule out potential false‐positive or false‐negative results, and to pay particular attention to the detection specificity in different population backgrounds.

Overall, the synergistic effect of BRAF V600E and TERT promoter mutations in PTC is not only closely related to the aggressiveness and recurrence rate of the tumor but may also influence patients’ therapeutic responses [46]. Therefore, in clinical practice, the detection and analysis of these two types of mutations are of great significance for the prognosis and treatment decision‐making of PTC patients [47]. Furthermore, elucidating the molecular mechanisms by which BRAF V600E mutations and TERT promoter mutations contribute to carcinogenesis, as well as how these alterations can be utilized for diagnostic and therapeutic purposes, is of great importance to both researchers and clinicians.

## 5. Conclusions

In conclusion, the ARMS‐qPCR assay provides a sensitive, specific, and reproducible tool for detecting BRAF and TERT promoter mutations in FFPE samples, outperforming Sanger sequencing in clinical scenarios requiring low‐abundance variant detection. Its integration into routine diagnostics could enhance patient stratification for targeted therapies in thyroid cancer and beyond.

## Author Contributions

Yuanyuan Jia and Min Gao conceived and designed the experiments. Dandan Sun, Jiabao Yang, and Mengshi Zhou performed the experiments and analyzed the data. Dandan Sun and Yuanyuan Jia wrote the original manuscript. Yajun Zhang provided clinical samples. Min Gao and Rui Li reviewed and edited the manuscript. Yuanyuan Jia and Yajun Zhang provided technical guidance. Min Gao and Rui Li conducted supervision.

## Funding

This research was funded by the Xi’an Science and Technology Plan Project “Key Core Technology Research Project for Priority Industrial Chains” (2023JH‐ZCGJ‐0071).

## Disclosure

All authors have read and agreed to the published version of the manuscript.

## Conflicts of Interest

The authors declare no conflicts of interest.

## Data Availability

Data are available upon request from the authors.
